# Supplementary material for: The interactome of intact mitochondria by cross-linking mass spectrometry provides evidence for coexisting respiratory supercomplexes
Source: Mol Cell Proteomics. 2017 Dec 8;17(2):216–32. doi: 10.1074/mcp.RA117.000470 (PMC5795388; doi:10.1074/mcp.RA117.000470)
Supplement: Supplemental Data [file supp_RA117.000470_133922_0_supp_23978_fzffwf.zip › spectra_annotation/mito_spectra_annotation/264-1-10-1-10-1.pdf]

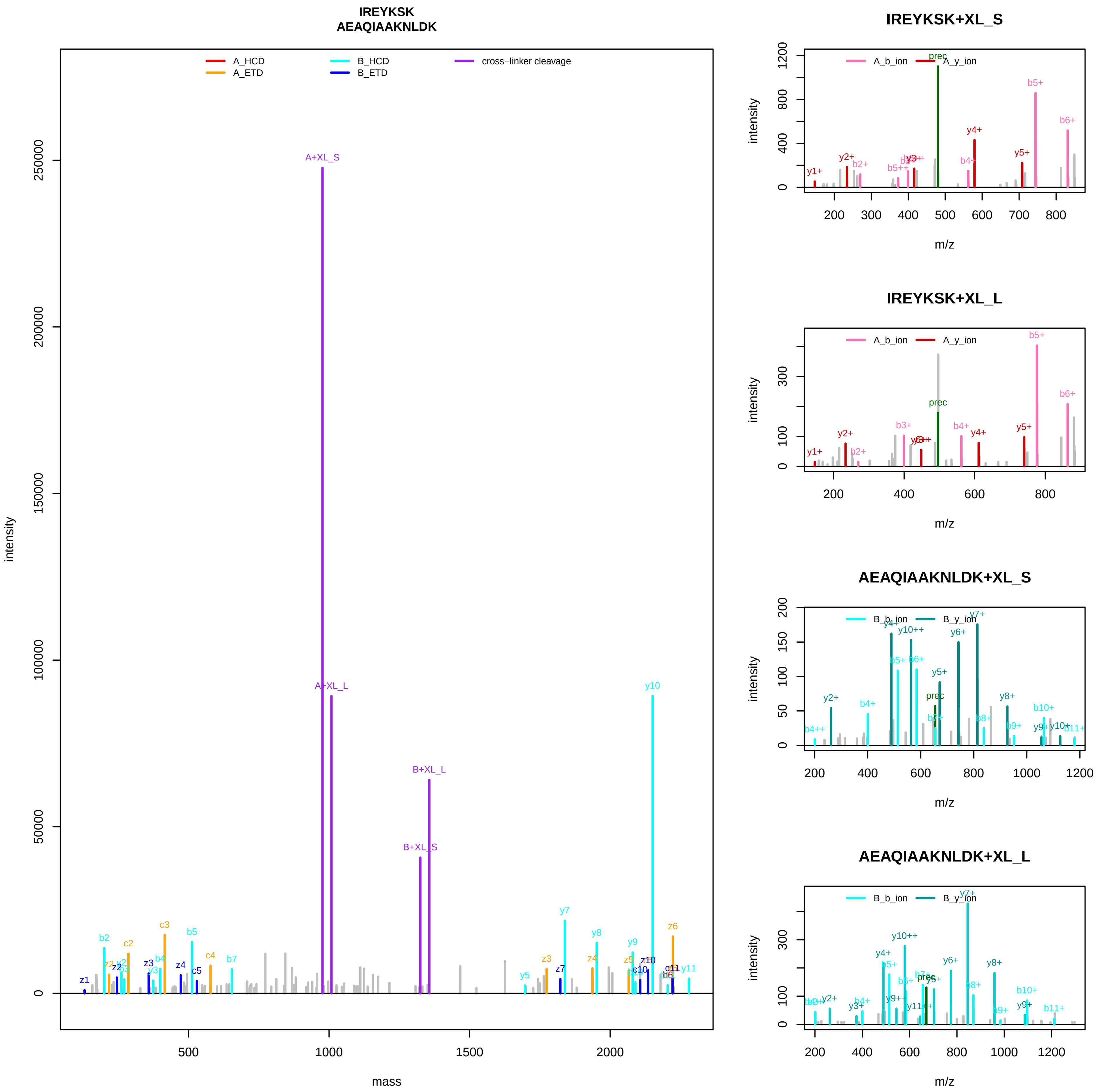

IREYKSK
AEAQIAAKNLDK
A_HCD
A_ETD
B_HCD
B_ETD
cross−linker cleavage
intensity
mass
0
50000
100000
150000
200000
250000
500
1000
1500
2000
A+XL_S
A+XL_L
B+XL_L
B+XL_S
y10
y7
y8
y9
z10
c10
c11
y11
z6
z5
z4
z3
z7
y5
b2
c2
c3
b5
c4
b7
b4
z1
z2
z3
z4
c5
y2
b3
y3
IREYKSK+XL_S
A_b_ion
A_y_ion
prec
b5+
b6+
y4+
y5+
y2+
b2+
y1+
b4+
b5++
intensity
m/z
0
400
800
1200
200
300
400
500
600
700
800
IREYKSK+XL_L
A_b_ion
A_y_ion
b5+
b6+
prec
b3+
b4+
y4+
y5+
y2+
y1+
b2+
0
100
300
200
400
600
800
intensity
m/z
AEAQIAAKNLDK+XL_S
B_b_ion
B_y_ion
y7+
y4+
y10++
y6+
b5+
b6+
y5+
prec
y2+
b4+
y8+
b10+
b4++
b7+
b8+
b9+
y9+
y10+
b11+
0
50
100
150
200
200
400
600
800
1000
1200
intensity
m/z
AEAQIAAKNLDK+XL_L
B_b_ion
B_y_ion
y7+
y10++
y4+
b5+
y6+
y8+
b7+
prec
y5+
b6+
b8+
y2+
b4+
y3+
y9++
y11++
b10+
y9+
b9+
b11+
0
100
300
200
400
600
800
1000
1200
intensity
m/z
